# Monocyte‐to‐Albumin Ratio Is Associated With Hematoma Expansion in Spontaneous Intracerebral Hemorrhage

**DOI:** 10.1002/brb3.70059

**Published:** 2024-09-30

**Authors:** Jie Fu, Yilin Xu, Xiu Chen, Jinglun Li, Lilei Peng

**Affiliations:** ^1^ Department of Neurology The Affiliated Hospital of Southwest Medical University Luzhou China; ^2^ Department of Neurosurgery The Affiliated Hospital of Southwest Medical University Luzhou China

**Keywords:** hematoma expansion, inflammation, intracerebral hemorrhage, monocyte‐to‐albumin ratio

## Abstract

**Background:**

Hematoma expansion (HE) after spontaneous intracerebral hemorrhage (ICH) is a severe complication that independently predicts poor prognosis. In this study, we aimed to investigate whether monocyte‐to‐albumin ratio (MAR), a novel marker of systemic inflammation, could predict HE in patients with ICH.

**Methods:**

We retrospectively assessed the data of patients with ICH. The clinical, imaging, and laboratory test data including, the MAR on admission, were analyzed. A multivariate logistic regression analysis was carried out to explore the relationship between MAR and hematoma growth. The receiver operating characteristic (ROC) curve was employed to investigate the predictive value of MAR for HE after ICH.

**Results:**

A total of 246 patients were included in the present study. Multivariate logistic regression analysis demonstrated that the MAR was associated with HE (odds ratio [OR] = 1.179; 95% confidence interval, 1.093–1.272; *p* = 0.000). ROC curve analysis showed that MAR could predict HE, with an area under the curve of 0.802 (95% CI: 0.744–0.859, *p *< 0.001). The optimal predictive cutoff value of MAR for HE was 10.01 (sensitivity: 72.43%, specificity: 77.05%).

**Conclusions:**

Our results suggested that a high MAR on admission was associated with an increased risk of HE in ICH patients, and MAR can become an independent predictor of HE in ICH patients.

## Graphical abstract

Our study aimed to investigate whether monocyte‐to‐albumin ratio (MAR), a novel marker of systemic inflammation, could predict hematoma expansion (HE) in patients with spontaneous intracerebral hemorrhage (ICH).

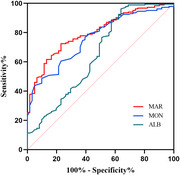

## 1 Introduction

Spontaneous intracerebral hemorrhage (ICH) is a lethal cerebrovascular disease, which is associated with high risks of disability and death, accounting for 10%–30% of all forms of stroke worldwide (Lattanzi et al. 2020). Hematoma enlargement (HE) is common at the early stage of patients with ICH. Around 73% of ICH patients suffered from increased hematoma volume during hospitalization, and most patients occurred 4–6 h after the onset of ICH (Wang et al. 2022). HE is a severe complication that independently predicts poor prognosis (Cao et al. 2017), and the risk of mortality was 5% higher for each 10% increase in hematoma volume (Wang et al. 2022). Therefore, predicting and preventing HE may be beneficial for improving the prognosis of ICH patients.

Prior studies have indicated that inflammation is associated with the pathophysiological processes of HE after ICH. A series of inflammatory responses occurs around the hematoma shortly following ICH (Chu et al. 2023), which could cause the breakdown of the blood–brain barrier (BBB), further induce constant blood vessel injury around primary hematoma location, and prolong bleeding (Li et al. 2020), ultimately contributing to HE.

Monocytes are important players of the innate immunity and play a critical role in regulating inflammation (Austermann, Roth, and Barczyk‐Kahlert 2022). Accumulating evidence indicates that monocytes are particularly critical for inflammatory injury following ICH. Hammond et al. (2014) found that in an experimental mouse ICH model, circulating inflammatory monocytes entered into the brain, outnumbered other leukocytes, and generated inflammatory cytokines. Furthermore, mice with fewer inflammatory monocytes had superior neurological function compared to controls. Additionally, another study retrospectively analyzed 186 patients with nontraumatic ICH, which revealed that baseline absolute monocyte count was related to higher 30‐day case fatality after ICH (Adeoye et al. 2014). Due to the lack of relation between absolute monocyte count and ICH volume, they speculated that absolute monocyte count may lead to post‐ICH secondary injury, including hematoma expansion and/or cerebral edema (Adeoye et al. 2014). Albumin is regarded as a negative acute‐phase protein, which declines during inflammation (Ishida et al. 2014). Low serum albumin levels have been reported to be related to poor functional outcomes in ICH patients (Limaye, Yang, and Hinduja 2016). Of note, the monocyte‐to‐albumin ratio (MAR), a novel marker of systemic inflammation, has been suggested to be involved in various diseases, including tumors (Zhao et al. 2023), cardiovascular diseases (Zhang et al. 2021), and hepatitis B virus‐associated decompensated cirrhosis (Yuan et al. 2023). Despite the fact that both monocytes and albumin are associated with outcomes and prognosis of ICH, the relationship between the MAR and HE in ICH patients remains unclear. Hence, the purpose of our study is to evaluate whether the MAR on admission could predict HE in ICH patients.

## 2 Materials and Methods

### 2.1 Study Population

We performed a retrospective review of patients with spontaneous ICH admitted to the affiliated hospital of Southwest Medical University from July 2021 to July 2023. The ethics committee of our hospital approved this study, and all patients or family members gave signed informed consent to take part in this investigation. The inclusion criteria included: (1) aged over 18 years old; (2) diagnosed with spontaneous ICH by noncontrast computed tomography (CT); and (3) patient received the baseline CT scan within 6 h after onset, and the second CT scan at 24 h after the first CT scan. Exclusion criteria included the following: (1) secondary ICH caused by brain trauma, aneurysm, arteriovenous malformation, moyamoya disease, brain tumor, or hemorrhagic transformation of acute cerebral infarction; (2) primary intraventricular hemorrhage (IVH); (3) patients underwent surgical evacuation before the follow‐up CT scan; (4) patients received anticoagulant or antiplatelet treatments before ICH onset; and (5) missing blood sample within 24 h after hospitalization.

### 2.2 Measurement of MAR

Monocyte count and albumin level on admission were obtained from the peripheral whole blood test. MAR was calculated as the ratio of monocyte count × 1000 to the albumin level.

### 2.3 Definition of HE

Two researchers (XYL and CX) independently assessed all the brain CT scans. Any discrepancy was resolved by negotiation or discussion with a third researcher (FJ). Hematoma volume was calculated by the ABC/2 method according to the previous description (Kothari et al. 1996). HE was defined as an absolute increase of hematoma of over 6 mL or a relative growth greater than 33% from the initial CT scan to the repeated CT scan (Demchuk et al. 2012).

### 2.4 Follow‐Up

The ICH patients discharged from the hospital were followed up by outpatient medical service or telephone interviewing, and 1‐year survival status was recorded.

### 2.5 Assessment of Covariates

Baseline clinical information was collected, including age, sex, time from symptom onset to the first CT scan, systolic and diastolic blood pressure on admission, Glasgow Coma Scale (GCS) score, past medical history of hypertension and diabetes, and current or previous history of smoking and drinking. Data of hematological covariates, including neutrophil count, lymphocyte count, monocyte count, red blood cell count, platelet count, prothrombin time, activated partial thromboplastin time, albumin, and blood glucose on admission, were collected. Radiological parameters were obtained, including ICH locations, hematoma volume, the presence of IVH, shape of hematoma, and density of hematoma.

### 2.6 Statistical Analysis

Normally distributed continuous variables were presented as means (standard deviation), and continuous variables with skewed distribution were expressed by medians (interquartile range), whereas categorical variables were described as frequency and percentage. The comparisons of clinical, imaging, and laboratory test data between ICH patients with and without HE (non‐HE) were performed by using the chi‐square (*χ* ^2^) test, the *t*‐test, or the Mann–Whitney *U*‐test as appropriate. Variables with *p* < 0.10 in the univariate analysis were selected to enter into the multivariate logistic regression model. Multicollinearity was evaluated with the variance inflation factor (VIF). The variable selected for further multivariate analysis was tolerance > 0.1 and VIF < 5. Receiver operating characteristic (ROC) curve analysis was applied to evaluate the predictive value of MAR for HE in ICH patients, and the DeLong test was used to compare the areas under ROC curves. The cutoff point of MAR was determined by the Youden index. Then, patients were classified into two groups by the value of MAR. The clinical and radiological data between the two groups were compared by applying the chi‐square (*χ* ^2^) test, the *t*‐test, or the Mann–Whitney *U*‐test. All statistical analyses were performed by using SPSS 26.0 and MedCalc 22.0 software, and *p* < 0.05 was regarded to be statistically significant.

## 3 Results

### 3.1 Comparison of Clinical Baseline Characteristics Between ICH Patients With and Without HE

**FIGURE 1:**
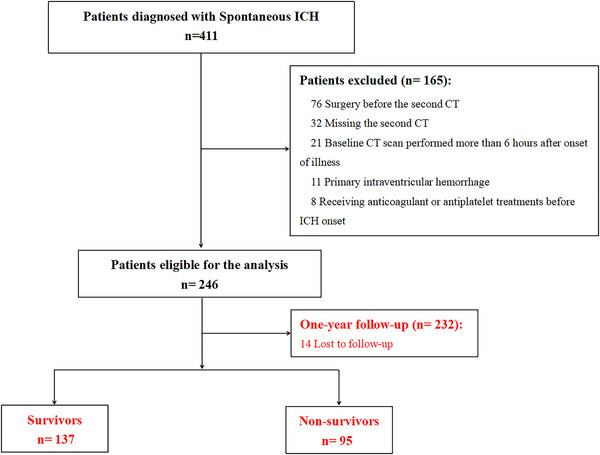
Flowchart of study enrollment. CT, computed tomography; ICH, intracerebral hemorrhage.

A total of 246 ICH patients were enrolled in our study (Figure 1). Patients were classified into two groups on the basis of HE presence. Clinical characteristics, as well as laboratory and imaging data of all patients, are shown and compared in Table 1. Among 246 ICH patients in our study, a total of 61 (24.8%) developed HE. The median MAR of the HE group was remarkably higher than that of the non‐HE group (*p* < 0.001). Additionally, compared to ICH patients without HE, patients with HE were more likely to have larger baseline hematoma volume, higher ratios of irregular hematoma and hematoma with mixed density, lower baseline albumin, higher baseline monocyte count, and blood glucose as well as prolonged prothrombin time and activated partial thromboplastin time (all *p* < 0.05). There were no significant differences in age, gender, medical history of hypertension and diabetes, personal smoking and drinking history, time to initial CT scan, blood pressure, GCS score, presence of IVH, hematoma location, neutrophil count, lymphocyte count, red blood cell count, and platelet count between ICH patients with and without HE (all *p* > 0.05).

**TABLE 1:** Univariate analysis of clinical characteristics and laboratory and imaging data between spontaneous intracerebral hemorrhage (ICH) patients with and without hematoma expansion.

	Hematoma expansion		*p* value
	Yes (*n *= 61)	No (*n *= 185)	
Age (years), mean (SD)	63.4 (14.0)	61.9 (11.5)	0.380
Male, *n* (%)	41 (67.2%)	108 (58.4%)	0.221
Hypertension, *n* (%)	47 (77.0%)	138 (74.6%)	0.700
Diabetes, *n* (%)	9 (14.8%)	15 (8.1%)	0.129
Current or previous smoking, *n* (%)	19 (31.1%)	43 (23.2%)	0.218
Current or previous drinking, *n* (%)	15 (24.6%)	39 (21.1%)	0.566
Onset‐to‐first‐CT time (hours), median (IQR)	3.5 (3.0–5.0)	4.0 (2.5–5.0)	0.615
SBP on admission (mmHg), mean (SD)	170.9 (24.8)	164.0 (24.5)	0.060
DBP on admission (mmHg), mean (SD)	97.0 (15.2)	94.9 (15.8)	0.362
GCS score, median (IQR)	13 (9–14)	13 (11–15)	0.123
Intraventricular hemorrhage, *n* (%)	17 (27.9%)	49 (26.5%)	0.833
Baseline hematoma volume (mL), median (IQR)	19.2 (16.2–27.4)	10.8 (6.3–18.9)	< 0.001
Hematoma location			0.703
Deep, *n* (%)	46 (75.4%)	133 (71.9%)	
Lobar, *n* (%)	9 (14.8%)	36 (19.5%)	
Infratentorial, *n* (%)	6 (9.8%)	16 (8.6%)	
Shape of hematoma			0.018
Regular, *n* (%)	26 (42.6%)	111 (60.0%)	
Irregular, *n* (%)	35 (57.4%)	74 (40.0%)	
Density of hematoma			0.018
Uniform density, *n* (%)	20 (32.8%)	93 (50.3%)	
Mixed density, *n* (%)	41 (67.2%)	92 (49.7%)	
RBC (× 10^12^/L), mean (SD)	4.58 (0.61)	4.50 (0.60)	0.389
NEU (× 10^9^/L), median (IQR)	6.29 (4.40–9.72)	6.83 (4.79–9.35)	0.528
LYM (× 10^9^/L), median (IQR)	1.12 (0.78–1.57)	1.05 (0.70–1.46)	0.206
MON (× 10^9^/L), median (IQR)	0.51 (0.39–0.65)	0.34 (0.25–0.45)	< 0.001
Platelet (× 10^9^/L), mean (SD)	205.1 (69.0)	200.7 (53.4)	0.603
PT (s), median (IQR)	11.9 (11.0–12.9)	11.4 (10.8–12.3)	0.043
APTT (s), median (IQR)	27.6 (24.8–31.2)	25.7 (23.8–29.1)	0.005
ALB (g/L), median (IQR)	40.8 (33.4–43.9)	42.8 (40.7–45.0)	< 0.001
MAR, median (IQR)	12.67 (10.07–17.42)	8.19 (5.76–10.66)	< 0.001
Blood glucose on admission (mmol/L), median (IQR)	7.4 (6.6–9.0)	7.0 (6.1–8.2)	0.033

Abbreviations: ALB, albumin; APTT, activated partial thromboplastin time; CT, computed tomography; DBP, diastolic blood pressure; GCS, Glasgow Coma Scale; IQR, interquartile range; LYM, lymphocyte count; MAR, monocyte‐to‐albumin ratio; MON, monocyte count; NEU, neutrophil count; PT, prothrombin time; RBC, red blood cell count; SBP, systolic blood pressure; SD, standard deviation.

### 3.2 Multivariate Logistic Regression Analysis

A multivariate logistic regression analysis was applied to investigate possible risk factors related to HE, which is shown in Table 2. The multivariate analysis demonstrated that MAR was independently related to HE (odds ratio [OR] = 1.179; 95% confidence interval, 1.093–1.272; *p* = 0.000). Additionally, systolic blood pressure on admission, baseline hematoma volume, and albumin on admission remained significant in the multivariate analysis (*p* = 0.009, *p* = 0.001, and *p* = 0.020, respectively).

**TABLE 2:** Multivariate analysis of predictors for hematoma expansion.

Variables	OR	95% CI	*p* value
SBP on admission	1.021	1.005–1.036	0.009
Baseline hematoma volume	1.061	1.024–1.098	0.001
PT	1.014	0.650–1.583	0.950
APTT	1.108	0.983–1.249	0.093
ALB	0.908	0.837–0.985	0.020
MAR	1.179	1.093–1.272	0.000
Blood glucose on admission	1.087	0.975–1.212	0.132
Shape of hematoma			
Regular	Reference	Reference	—
Irregular	1.222	0.530–2.819	0.638
Density of hematoma			
Uniform density	Reference	Reference	—
Mixed density	1.487	0.631–3.507	0.364

Abbreviations: ALB, albumin; APTT, activated partial thromboplastin time; CI, confidence intervals; MAR, monocyte‐to‐albumin ratio; OR, odds ratio; PT, prothrombin time; SBP, systolic blood pressure.

### 3.3 The Predictive Power of MAR for HE

**FIGURE 2:**
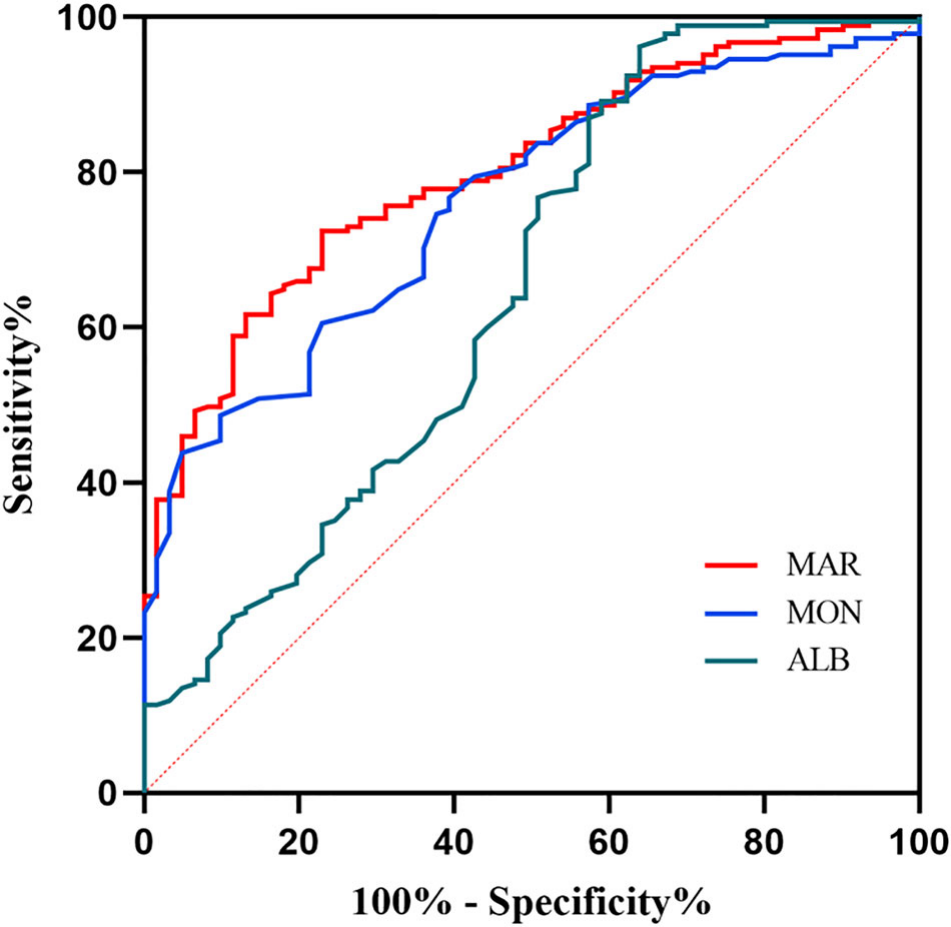
Receiver operating characteristic curve of MAR, albumin (ALB), and monocyte (MON) to predict HE in ICH patients. HE, hematoma expansion; ICH, intracerebral hemorrhage; MAR, monocyte‐to‐albumin ratio.

We employed the ROC curve to explore the predictive ability of MAR for HE in ICH patients. The area under the ROC curve of MAR was 0.802 (95% CI: 0.744–0.859, *p* < 0.001) for HE, which was larger than those of monocyte count (0.761, 95% CI: 0.698–0.824, *p* < 0.001) and albumin (0.651, 95% CI: 0.564–0.738, *p* < 0.001) (Figure 2), which reached statistical significance (difference between areas MAR vs. monocyte count: 0.038, 95% CI: 0.010–0.066, *p* = 0.008; difference between areas MAR vs. albumin: 0.148, 95% CI: 0.058–0.238, *p* = 0.001). The optimal predictive cutoff value for HE by MAR was 10.01 (sensitivity 72.43%, specificity 77.05%). Furthermore, all ICH patients were classified into two groups on the basis of the cutoff point of MAR (MAR < 10.01 and MAR ≥ 10.01). We compared the clinical and imaging variables between the two groups (Table 3). As expected, HE was more common in patients with MAR ≥ 10.01 than those with MAR < 10.01 (*p* < 0.001). Additionally, we evaluated the association between MAR and 1‐year mortality in ICH patients. Follow‐up data were available for 232 patients (Figure 1), and we also divided these patients into two groups the basis of the value of MAR (MAR < 10.01 and MAR ≥ 10.01). Our results indicated that compared to ICH patients with MAR < 10.01, a higher ratio of 1‐year mortality was observed in ICH patients with MAR ≥ 10.01 (*p* < 0.001) (Table 3). Moreover, compared to ICH patients with MAR < 10.01, greater baseline hematoma volume and higher male ratio were observed in ICH patients with MAR ≥ 10.01 (*p* = 0.006 and *p* = 0.010, respectively). No obvious differences were found in the age, smoking, drinking, medical history of hypertension and diabetes, blood pressure, GCS score, IVH, time to initial CT scan, ICH locations, shape of hematoma, and density of hematoma between patients with MAR ≥ 10.01 and < 10.01 (all *p* > 0.05).

**TABLE 3:** Comparison of baseline demographic and radiological characteristics between patients with different levels of monocyte‐to‐albumin ratio (MAR).

	MAR		*p* value
	< 10.01 (*n* = 148)	≥ 10.01 (*n* = 98)	
Age (years), mean (SD)	61.8 (12.4)	62.9 (11.9)	0.466
Male, *n* (%)	80 (54.1%)	69 (70.4%)	0.010
Hypertension, *n* (%)	106 (71.6%)	79 (80.6%)	0.110
Diabetes, *n* (%)	12 (8.1%)	12 (12.2%)	0.284
Current or previous smoking, *n* (%)	34 (23.0%)	28 (28.6%)	0.322
Current or previous drinking, *n* (%)	31 (20.9%)	23 (23.5%)	0.640
Onset‐to‐first‐CT time (hours), median (IQR)	4 (3–5)	4 (2–5)	0.857
SBP on admission (mmHg), mean (SD)	165 (25)	166 (24)	0.767
DBP on admission (mmHg), mean (SD)	96 (16)	95 (14)	0.641
GCS score, median (IQR)	13 (12–15)	13 (10–15)	0.089
Intraventricular hemorrhage, *n* (%)	42 (28.4%)	24 (24.5%)	0.500
Baseline hematoma volume (mL), median (IQR)	11.6 (7.5–18.9)	16.8 (8.8–25.4)	0.006
Hematoma location			0.389
Deep, *n* (%)	105 (70.9%)	74 (75.5%)	
Lobar, *n* (%)	31 (21.0%)	14 (14.3%)	
Infratentorial, *n* (%)	12 (8.1%)	10 (10.2%)	
Shape of hematoma			0.499
Regular, *n* (%)	85 (57.4%)	52 (53.1%)	
Irregular, *n* (%)	63 (42.6%)	46 (46.9%)	
Density of hematoma			0.067
Uniform density, *n* (%)	75 (50.7%)	38 (38.8%)	
Mixed density, *n* (%)	73 (49.3%)	60 (61.2%)	
Hematoma expansion, *n* (%)	14 (9.5%)	47 (48.0%)	< 0.001
One‐year mortality (data available for 138/94 patients), *n* (%)	39 (28.3%)	56 (59.6%)	< 0.001

Abbreviations: CT, computed tomography; DBP, diastolic blood pressure; GCS, Glasgow Coma Scale; IQR, interquartile range; SBP, systolic blood pressure; SD, standard deviation.

## 4 Discussion

Our study explored the predictive power of MAR for HE in ICH patients, which indicated that a high MAR on admission was closely associated with early HE after ICH. In addition, MAR on admission may be also related to 1‐year mortality in ICH patients. Of note, this study for the first time investigated the correlation between the MAR and HE after ICH.

The occurrence of HE within 6 h from symptom onset ranges from 13% to 38% in ICH patients (Li et al. 2020). Following ICH, the inflammatory cascade of the central nervous system is induced by activated glial cells, leading to the release of pro‐inflammatory factors around the hematoma region (Tschoe et al. 2020). Moreover, the clots caused by the vessel rupture could induce the production of thrombin, and thrombin has been indicated to be able to activate the inflammatory responses and the expression of matrix metalloproteinase (MMP) (Chen et al. 2017). MMPs lead to BBB damage and the upregulation of cell adhesion molecules around the hematoma, which contributes to the recruitment of systemic leukocytes (Magid‐Bernstein et al. 2022). Furthermore, these systemic immune cells are activated and then generate extra pro‐inflammatory factors (Magid‐Bernstesin et al. 2022). Inflammatory state could destroy coagulation function and vessel wall integrity, which results in continuous leakage of vessels, as well as subsequent HE formation (Chen et al. 2017). Therefore, inflammation is suggested to be a major factor of HE after ICH (Wang et al. 2022), and various inflammatory markers, such as CRP, IL‐10, and IL‐6, have been evidenced to be closely related to HE and hold predictive value for HE in ICH (Di Napoli et al. 2014; Silva et al. 2005; Wang et al. 2011).

The white blood cell count, as a marker of inflammation, is generally available in clinical practice, and the relationship between white blood cell as well as its subsets and early HE following ICH has been widely investigated. In accordance with previous studies (Morotti et al. 2016), our study also found that higher monocyte count on admission was related to an increased risk of HE. It is reported that peripheral blood mononuclear cell (PBMC) gene expression was changed within 6 h of ICH onset, which was associated with monocyte activation (Walsh et al. 2019). As the monocyte surface contains strong physiologic anticoagulants, monocyte activation could hinder clot generation and fibrin stabilization and thus facilitate subsequent bleeding (Morotti et al. 2016). Moreover, monocyte activation could be associated with elevated pro‐inflammatory cytokine production (Allali et al. 2022), which may further damage BBB and thus cause brain edema and HE occurrence (Adeoye et al. 2014). However, the relationship between HE after ICH and lymphocyte count remains controversial. Our study observed no obvious difference in lymphocyte count between ICH patients with and without HE, which was in‐line with two previous investigations (Alimohammadi et al. 2022; Morotti et al. 2016). In contrast, the study by Li et al. (2023) indicated a remarkable association between HE and baseline lymphocyte counts. One possible explanation for the paradoxical results may be that ICH patients with different ages were included. In our study and the two prior investigations, the mean age of included ICH patients was more than 60 years old, whereas the mean age was below 60 years old in the study by Li et al. Of note, a previous research indicated the age‐dependent variation in the absolute lymphocyte count (MacKinney 1978). Thus, we speculate that the difference in the lymphocyte count between ICH patients with and without HE may be also age‐dependent, which needs to be clarified by the future studies.

As a negative acute‐phase protein, albumin has been suggested to hold the anti‐inflammatory and antioxidant properties (Deng et al. 2021; Wiedermann 2007). Under the inflammatory state, albumin levels are reduced due to decreased hepatic synthesis and increased capillary leakage (Zhang et al. 2021). Previous investigations have revealed the potential correlation between albumin levels and prognosis of ICH patients. Limaye, Yang, and Hinduja (2016) reported that reduced albumin levels on admission were related to poor clinical outcomes in ICH patients. Moreover, albumin can maintain BBB integrity and reduce neuronal oxidative stress and apoptosis, thus providing neuroprotection and contributing to functional recovery after ICH (Cao and Yao 2024). Thus, without albumin protection, ICH patients with low levels of albumin are more likely to encounter HE.

According to the above, the MAR, a ratio of monocyte and albumin, may be a potential and stable predictor of HE after ICH. However, no relevant studies have been conducted to explore the possibility of MAR for predicting HE, which leaves a major knowledge gap. Fortunately, in the present study, we observed a remarkable correlation between the MAR and HE, and a high MAR level on admission was an effective predictor for early HE in ICH patients. Our results suggest that the MAR could become a novel and effective blood biomarker for predicting the risk of HE in ICH patients.

In addition, we observed that a greater baseline hematoma volume and a higher systolic blood pressure were associated with HE in our study, which were in‐line with several previous studies (Li et al. 2023; Ohwaki et al. 2004). ICH patients with post‐admission systolic blood pressure more than 160 mmHg are more likely to suffer from HE, which could result from the constant damage and bleeding of small vessels (Li et al. 2020). Baseline ICH volume has been suggested to be associated with the risk of HE. Large hematomas (> 30 mL) are more likely to expand, whereas small ICH volume (< 10 mL) is less likely to relate to HE (Dowlatshahi, Demchuk, et al. 2011). A possible reason is that greater baseline ICH volume may cause vessel injury and multifocal bleeding due to mass effect and vessel shearing, further leading to perilesional hemorrhage and HE formation (Dowlatshahi, Smith, et al. 2011).

ICH is an important public health problem, which is associated with high risks of disability and death in adults (Qureshi, Mendelow, and Hanley 2009). Given HE being associated with worse outcomes of ICH, early recognition of HE is vital to take timely preventive measures and improve the functional outcomes and prognosis of ICH patients. Our study identified the clinical implications of MAR for predicting HE in ICH patients. First, MAR, as a routine index of blood testing, is easily available in clinical practice. Second, MAR consists of two inflammation‐related indexes, monocyte count and albumin level, which could result in more reliable results. Additionally, considering the limitation of a single indicator for predicting HE, the combination of MAR and other predictors could be investigated to generate new prediction scores to increase the predictive specificity and sensitivity.

Several limitations were noted in the present study. First, this was a single‐center retrospective study. Second, this study has a relatively small sample size. Third, although the multivariate analysis was employed, potential confounding factors may still affect the results of our study.

## 5 Conclusion

This study indicated that a high MAR level on admission was closely associated with HE after ICH. Our findings support that MAR may become an effective biomarker to recognize ICH patients at higher risk of HE, assisting clinicians to take adequate and individual therapies. Considering the limitations of the present study, future more well‐designed investigations with larger sample size are needed to warrant our findings.

## 6 Clinical Implications

Hematoma enlargement (HE) is a severe complication that independently predicts poor prognosis of ICH. The results of our study indicated that MAR, a novel marker of systemic inflammation, could predict HE in patients with ICH. ICH patients with a high MAR on admission may face an increased risk of HE, and thus early detection of MAR is necessary, in order to carry out timely interventions and improve the prognosis of ICH patients.

## Author Contributions

**Jie Fu**: conceptualization, investigation, funding acquisition, writing–original draft, software. **Yilin Xu**: investigation, methodology, formal analysis. **Xiu Chen**: investigation, validation. **Jinglun Li**: investigation, methodology. **Lilei Peng**: conceptualization, writing–review and editing, supervision.

## Conflicts of Interest

The authors declare no conflicts of interest.

### Peer Review

The peer review history for this article is available at https://publons.com/publon/10.1002/brb3.70059.

## Data Availability

The data used to support the findings of this study are included within the article.
